# Supplementary material for: Towards exact molecular dynamics simulations with machine-learned force fields
Source: Nat Commun. 2018 Sep 24;9:3887. doi: 10.1038/s41467-018-06169-2 (PMC6155327; doi:10.1038/s41467-018-06169-2)
Supplement: Supplementary file 1 — Supplementary Information [file 41467_2018_6169_MOESM1_ESM.pdf]

Towards Exact Molecular Dynamics Simulations with Machine-Learned Force Fields

Supplementary Information

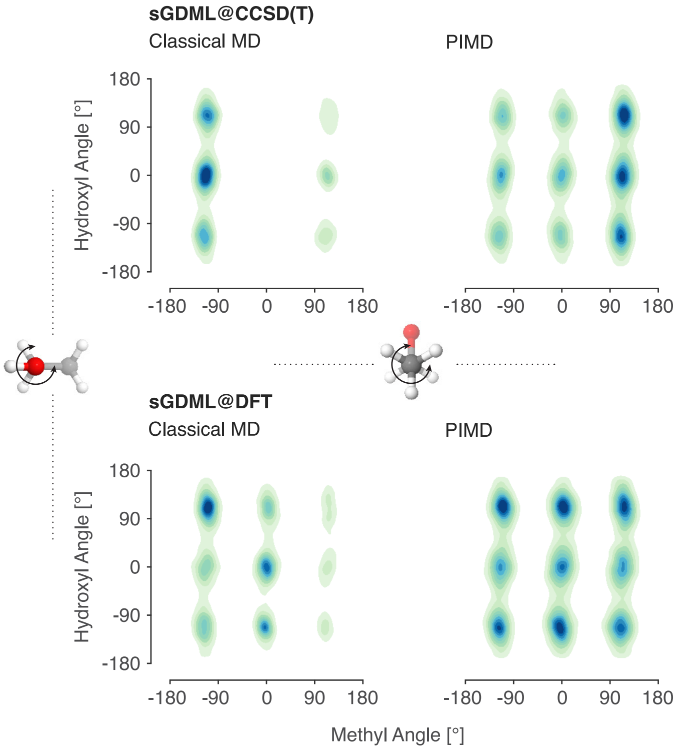

Supplementary Figure 1. Comparison of probability distributions of the dihedral angles (methyl rotor vs. hydroxyl rotor) of ethanol obtained from classical and path-integral MD simulations at 300 K. We contrast the results from a sGDML model trained on CCSD(T) versus DFT reference calculations. The inclusion of nuclear quantum effects improves the sampling of the PES for both levels of theory. The sampling was performed during 0.5 ns of simulation, using 16 beads for PIMD.

Supplementary Table 1. Prediction accuracy for interatomic forces and total energies of the sGDML@DFT on all datasets. Energy errors are in kcal mol^{-1}, force errors in kcal mol^{-1}Å^{-1}.

Dataset		Energy Prediction		Force Prediction					
						Magnitude		Angle	
Molecule	# ref.	MAE	RMSE	MAE	RMSE	MAE	RMSE	MAE	RMSE
Benzene	1000	0.10	0.12	0.06	0.09	0.06	0.09	0.0009	0.0017
Uracil	1000	0.11	0.14	0.24	0.37	0.22	0.31	0.0039	0.0064
Naphthalene	1000	0.12	0.15	0.11	0.17	0.11	0.15	0.0016	0.0026
Aspirin	1000	0.19	0.25	0.68	0.96	0.52	0.68	0.0094	0.0139
Salicylic acid	1000	0.12	0.15	0.28	0.44	0.32	0.45	0.0038	0.0064
Malonaldehyde	1000	0.10	0.13	0.41	0.62	0.39	0.56	0.0055	0.0087
Ethanol	1000	0.07	0.09	0.33	0.49	0.46	0.63	0.0051	0.0083
Toluene	1000	0.10	0.12	0.14	0.21	0.14	0.19	0.0020	0.0031
Paracetamol	1000	0.15	0.20	0.49	0.70	0.60	0.84	0.0073	0.0118
Azobenzene	1000	0.09	0.13	0.41	0.61	0.49	0.71	0.0059	0.0105

Supplementary Table 2. Prediction accuracy for interatomic forces and total energies of the sGDML@CCSD(T) model on all datasets. Energy errors are in kcal mol^{-1}, force errors in kcal mol^{-1}Å^{-1}.

Dataset		Energy Prediction		Force Prediction					
						Magnitude		Angle	
Molecule	# ref.	MAE	RMSE	MAE	RMSE	MAE	RMSE	MAE	RMSE
Benzene	1000	0.004	0.005	0.04	0.06	0.04	0.06	0.0008	0.0013
Aspirin*	1000	0.16	0.21	0.76	1.07	0.56	0.74	0.0091	0.0123
Malonaldehyde	1000	0.06	0.08	0.37	0.56	0.34	0.46	0.0052	0.0082
Ethanol	1000	0.05	0.07	0.35	0.51	0.47	0.65	0.0056	0.0104
Toluene	1000	0.03	0.04	0.21	0.30	0.19	0.24	0.0028	0.0042

* CCSD

Supplementary Note 1. Alternative symmetry-adapted kernels

Assignment kernel

As an alternative to symmetrizing the kernel function, it is conceivable to normalize the data to a canonical permutational configuration instead. Such an *assignment kernel function* performs a local reconstruction of the symmetric part of the target function which is then "tiled" across the entire input domain. This involves compressing the data $\mathbf{x}_i$ to one of its symmetric subdomains via transformation to a fixed canonical reference configuration $\mathbf{P}_{i1}\mathbf{x}_i$ prior to training. Such an approach bears two major disadvantages over our symmetric model:

- Every query molecule must be first matched to the training set, making evaluations of the model computationally costly.

- The "tiling" process causes discontinuous seams to form along borders of neighboring symmetric subdomains, were different copies of the local model meet. These seams correspond with the symmetry lines of the molecule, which are frequently crossed during MD simulations. Moreover, they reside in the extrapolation regime of the local model, where the prediction performance is notoriously bad.

Our model resolves these issues by effectively optimizing all symmetric subdomains simultaneously. It retains all advantages of the assignment kernel.

R-convolution kernel

A generic design paradigm for comparing complex structured objects like molecules, is the R-convolution kernel [1]. It partitions the object into smaller components $\mathbf{x} = (\mathbf{x}_1, \ldots, \mathbf{x}_D)$ for which a similarity measure in terms of a kernel function $k_d \colon \mathcal{X} \times \mathcal{X} \to \mathbb{R}$ is easier to define. The sum over all possible component assignments

$$\kappa(\mathbf{x}, \mathbf{x}') = \sum_{\substack{\text{parts}(\mathbf{x}) \\ \text{parts}(\mathbf{x}')}} \prod_{d}^{D} \kappa_d(\mathbf{x}_d, \mathbf{x}'_d) \tag{1}$$

gives an approximation of the overall similarity of both objects, which – by the closure properties of kernels [2, 3] – is again a valid kernel. Most permutation invariant models rely on instances of this idea and only differ by the underlying partitioning scheme and similarity measure to compare the components (e.g. graph kernels compare paths [4]). The combinatorial nature of this approach guarantees that the correct assignments are always captured, alas only in unison with all the others. This imprecision inevitably limits the sensitivity of such a metric. Our kernel function explicitly exercises the correct symmetries and is hence able to provide unbiased similarities.

Supplementary Note 2. Permutation matrices

Throughout this paper, we use permutation matrices $\mathbf{P}(\tau) \equiv \mathbf{P}$ in column representation, obtained by permuting the columns of the identity matrix of dimension $N \times N$, such that $(\mathbf{P})_{ij} = 1$ if $j = \tau(i)$ and 0 otherwise. The multiplication $\mathbf{Px}$ will hence permute the rows of the column vector $\mathbf{x}$. We do not distinguish between permutation matrices acting on different representations of the same data. While $\mathbf{PR}^\top$ permutes the atoms of a molecule represented by a $3 \times N$ matrix $\mathbf{R} = (\mathbf{r}_1, \ldots, \mathbf{r}_N)$ of Cartesian coordinates, $\mathbf{P}^{(\mathcal{I})}$ represents the same permutation, but acting on a linearized input space $\mathcal{I}$ representation $\mathbf{R}^{(\mathcal{I})} = (\mathbf{r}_1^\top, \ldots, \mathbf{r}_N^\top)^\top$ of dimension $3N \times 1$.

Supplementary Note 3. Training sGDML using a descriptor

For notational convenience, the main text describes the formulation of the sGDML model for generic inputs $\mathbf{x}$. When the input to the force field kernel function is a descriptor, the symmetric (training) kernel matrix evaluates to

$$\text{Hess}(\kappa_{\text{sym}})(\mathbf{D}(\mathbf{x}), \mathbf{D}(\mathbf{x}')) = \frac{1}{S} \sum_{pq}^{S} (\nabla \mathbf{D}(\mathbf{P}_p \mathbf{x}) \mathbf{P}_p)^\top \text{Hess}(\kappa)(\mathbf{D}(\mathbf{P}_p \mathbf{x}), \mathbf{D}(\mathbf{P}_q \mathbf{x}')) \nabla \mathbf{D}(\mathbf{P}_q \mathbf{x}) \mathbf{P}_q \tag{2}$$

after application of the chain rule, where $\nabla \mathbf{D}$ is the gradient of the descriptor.

SUPPLEMENTARY REFERENCES

[1] Haussler, D. Convolution kernels on discrete structures. Technical Report, University of California at Santa Cruz, Santa Cruz, CA, USA (1999).

[2] Schölkopf, B. & Smola, A. J. *Learning with Kernels: Support Vector Machines, Regularization, Optimization, and Beyond* (MIT Press, Cambridge, MA, USA, 2002).

[3] Müller, K.-R., Mika, S., Ratsch, G., Tsuda, K. & Scholkopf, B. An introduction to kernel-based learning algorithms. *IEEE Trans. Neural Netw.* **12**, 181–201 (2001).

[4] Vishwanathan, S. V. N., Schraudolph, N. N., Kondor, R. & Borgwardt, K. M. Graph kernels. *J. Mach. Learn. Res.* **11**, 1201–1242 (2010).
